# Predicting High-Grade Cancer at Ten-Core Prostate Biopsy Using Four Kallikrein Markers Measured in Blood in the ProtecT Study

**DOI:** 10.1093/jnci/djv095

**Published:** 2015-04-11

**Authors:** Richard J. Bryant, Daniel D. Sjoberg, Andrew J. Vickers, Mary C. Robinson, Rajeev Kumar, Luke Marsden, Michael Davis, Peter T. Scardino, Jenny Donovan, David E. Neal, Hans Lilja, Freddie C. Hamdy

**Affiliations:** **Affiliations of authors:**Nuffield Department of Surgical Sciences, University of Oxford, UK (RJB, RK, LM, HL, FCH); Department of Epidemiology & Biostatistics, Memorial Sloan Kettering Cancer Center, New York, NY (DDS, AJV); Department of Cellular Pathology, Royal Victoria Infirmary, Newcastle upon Tyne, UK (MCR); School of Social and Community Medicine, University of Bristol, UK (MD); Department of Surgery, Urology Service, Memorial Sloan Kettering Cancer Center (PTS, HL); Department of Oncology, University of Cambridge, UK (DEN); Departments of Laboratory Medicine (Clinical Chemistry Service) and Medicine (Genitourinary Oncology Service), Memorial Sloan Kettering Cancer Center, New York, NY (HL); Department of Laboratory Medicine and Clinical Sciences in Malmö, Lund University, Skåne University Hospital, Malmö, Sweden; and Institute of Biomedical Technology, University of Tampere, Finland (HL).

## Abstract

**Background:**

Many men with elevated prostate-specific antigen (PSA) levels in serum do not have aggressive prostate cancer and undergo unnecessary biopsy. Retrospective studies using cryopreserved serum suggest that four kallikrein markers can predict biopsy outcome.

**Methods:**

Free, intact and total PSA, and kallikrein-related peptidase 2 were measured in cryopreserved blood from 6129 men with elevated PSA (≥3.0ng/mL) participating in the prospective, randomized trial Prostate Testing for Cancer and Treatment. Marker levels from 4765 men providing anticoagulated plasma were incorporated into statistical models to predict any-grade and high-grade (Gleason score ≥7) prostate cancer at 10-core biopsy. The models were corrected for optimism by 10-fold cross validation and independently validated using markers measured in serum from 1364 men. All statistical tests were two-sided.

**Results:**

The four kallikreins enhanced prostate cancer detection compared with PSA and age alone. Area under the curve (AUC) for the four kallikreins was 0.719 (95% confidence interval [CI] = 0.704 to 0.734) vs 0.634 (95% CI = 0.617 to 0.651, *P* < .001) for PSA and age alone for any-grade cancer, and 0.820 (95% CI = 0.802 to 0.838) vs 0.738 (95% CI = 0.716 to 0.761) for high-grade cancer. Using a 6% risk of high-grade cancer as an illustrative cutoff, for 1000 biopsied men with PSA levels of 3.0ng/mL or higher, the model would reduce the need for biopsy in 428 men, detect 119 high-grade cancers, and delay diagnosis of 14 of 133 high-grade cancers. Models exhibited excellent discrimination on independent validation among men with only serum samples available for analysis.

**Conclusions:**

A statistical model based on kallikrein markers was validated in a large prospective study and reduces unnecessary biopsies while delaying diagnosis of high-grade cancers in few men.

Risk of death from prostate cancer is strongly associated with levels of prostate-specific antigen (PSA) in blood measured in middle-aged men (1). Evidence from randomized screening trials in Europe shows that PSA-based screening can reduce deaths from prostate cancer (2–4), but also leads to overdiagnosis and the risk of overtreatment among elderly men with a limited life expectancy (5,6). Although the PSA test detects an increased risk of prostate cancer at an early stage of the disease, it has low specificity (7) such that most men with an elevated PSA either do not have prostate cancer or have low-risk disease that is unlikely to affect quality or length of life if left untreated. An elevated PSA is the main indication for the approximately one million prostate biopsies performed per annum in the United States (8). Annual prostate cancer incidence in the United States is close to 250 000, illustrating the unmet need for markers that contribute specificity beyond that of total PSA in order to discriminate between men with cancers likely to influence the length or quality of life and those with indolent disease or benign conditions associated with PSA elevation in blood.

Previous research suggested that a panel of free PSA (fPSA), “intact” PSA (iPSA—detecting only noncatalytic single-chain free PSA but not multichain-free PSA internally cleaved between Lys_145_ or Lys_146_ [9]), and total PSA (tPSA), as well as human kallikrein–related peptidase 2 (hK2) measured in blood, is more accurate in predicting the outcome of prostate biopsy than total PSA alone among previously unscreened (10–12) and previously screened (11,12) men and men with a previous negative biopsy (13). Decision analyses showed that a statistical model based on the four kallikrein markers in blood can improve clinical decision-making about biopsy for men with a PSA above 3ng/mL (10,11,14). These data suggest that the number of men undergoing biopsy could be reduced to half using 20% or greater cancer risk as a tentative threshold for biopsy, with approximately 20% of cancers remaining undetected among previously unscreened men. However, most of these cancers would be low-grade and low-stage cancers typically associated with overdiagnosis, while few high-grade cancers would be missed.

The Prostate Testing for Cancer and Treatment (ProtecT) study in the United Kingdom is a prospective randomized controlled trial evaluating the cost-effectiveness of conventional treatment modalities in PSA-detected clinically localized prostate cancer. A total of 8565 of 82 428 (10.4%) men recruited to ProtecT had a PSA of 3.0ng/mL or greater and were offered a standard 10-core prostate biopsy. Of the 7413 participants receiving biopsies, 2894 were found to have evidence of prostate cancer (15,16). In previous studies, serum samples had been used to measure the four kallikrein markers. However, it is well recognized that ethylenediaminetetraacetic acid (EDTA) anticoagulated plasma has advantages over serum with free and intact PSA, being less prone to degradation in plasma, enabling more accurate biomarker analyses with samples shipped to laboratories distant from the point of care (9,17). The majority of participants enrolled in ProtecT had EDTA anticoagulated blood collected, which provided unique opportunities to compare the kallikrein markers measured in plasma vs serum. Previous evaluations of the four kallikrein markers were limited to men undergoing sextant prostate biopsies (10–13), while the study reported herein has the added value of assessing the markers in the more contemporary extended 10-core biopsy protocol used in ProtecT (15), as this leads to higher rates of cancer detection (18,19). We performed retrospective measurements of four kallikrein markers (tPSA, fPSA, iPSA, and hK2) in cryopreserved EDTA anticoagulated blood or serum in the context of a large randomized prospective clinical trial involving contemporary extended 10-core biopsies to determine whether this panel of markers improves prediction of biopsy outcomes compared with total PSA and age.

## Methods

### Patient Cohort

In the ProtecT study, 228 926 men aged 50 to 69 years were invited between 2001 and 2008 to receive PSA testing. Of those, 82 429 (36%) participants were tested, 8565 men with a serum PSA measurement of 3.0ng/mL or greater were invited to undergo a 10-core prostate biopsy, 7471 (87%) men were biopsied, and prostate cancer was detected in 2637 participants. Two men with missing age information at biopsy and three men with missing Gleason grading were omitted from all analyses. Cryopreserved blood was retrieved for 82% of biopsied ProtecT participants: EDTA anticoagulated plasma from 4765 men, serum from 1860 men, and both plasma and serum from 496 men. Participants had consented to sample collection and analysis by enrollment in the Prostate Cancer Mechanisms of Progression and Treatment (ProMPT) study (ethics approval NRES 01/04/061).

### Pathological Analysis

Assessment of biopsies was undertaken blind to marker results by urological pathologists using standardized protocols and agreed-upon reporting proformas (20), cancers were graded using the standard Gleason system (21), as detailed in a recent publication providing information on the processing and reporting of prostate cores and changes in Gleason scores over time (22).

### Laboratory Methods

For consented individuals undergoing PSA testing within the ProtecT study, blood was collected, centrifuged, and frozen at -80° C. Anticoagulated plasma or serum was obtained after centrifugation at 3000 *g* for 10 minutes within 30 to 60 minutes of venipuncture. Immunoassays for fPSA, tPSA (17,23), iPSA, and hK2 (24,25) were performed as previously reported (11) using cryopreserved samples shipped on dry ice for analysis in Hans Lilja’s laboratory at the Wallenberg Research Laboratories, Department of Laboratory Medicine, Lund University, Skåne University Hospital, Malmö, Sweden. Sample aliquots were subject to two freeze-thaw cycles, and analyses were performed with researchers blinded to prostate biopsy outcome.

### Statistical Methods

Previous statistical models were based on kallikrein levels measured in serum for previously unscreened men undergoing sextant prostate biopsy as part of the European Randomized Study of Screening for Prostate Cancer (ERSPC) study in Rotterdam (10,11). As levels of some of the kallikrein markers differ in anticoagulated plasma vs serum and the biopsied men in the ProtecT cohort were subjected to extended 10-core biopsy, we generated new prediction models. We used multivariable logistic regression to build models predicting presence of any-grade or high-grade disease on biopsy based on a man’s age, tPSA, fPSA, iPSA, and hK2. Restricted cubic splines with knots at the tertiles were included in the model for tPSA and fPSA but not iPSA and hK2. Predictions for men with a PSA level of greater than 25ng/mL were based on tPSA levels alone. The use of splines for tPSA and fPSA, but not iPSA or hK2, and the use of a 25ng/mL PSA cutoff were predetermined based on our prior research, and the only model fitting was to estimate an intercept and coefficients for each marker and nonlinear term. We report the area under the curve (AUC), or discrimination, of the newly developed models. AUCs were compared with models based on standard clinically available predictors using the DeLong test (26). We also investigated whether the model based on the four-kallikrein panel could reduce the number of men undergoing biopsy without delaying the diagnosis of high-grade disease in many men. We used decision curve analysis (27) to investigate the potential clinical effects of our models. All reported statistics based on modelling were corrected for overfit using 10-fold cross-validation. As serum samples were available for some men in the cohort, we conducted exploratory analyses applying our previously developed models to this subgroup. All *P* values reported are two-sided. Analyses were conducted using Stata 12.0 (StataCorp., College Station, TX).

## Results

Characteristics of the 6129 biopsied men with blood samples are shown in Table 1. Anticoagulated plasma (primary analysis) was available from 4765 men (3032 with no cancer, 1733 with cancer), and serum (secondary analysis) was available from another 1860 men (1221 with no cancer and 639 with cancer). Finally, we measured the four kallikrein markers in aliquots from 496 men who had both plasma and serum samples (Supplementary Table 1, available online). Overall, prior PSA testing was low among biopsied men (18% for men providing plasma and 16% for men providing serum).

**Table 1.** Characteristics of men in the ProtecT study cohort*

	EDTA anticoagulated blood plasma			Serum		
Characteristics	No cancer detected n = 3032, 64% No. (%)	Diagnosed with cancer n = 1733, 36% No. (%)	*P*	No cancer detected n = 1221, 66%No. (%)	Diagnosed with cancer n = 639, 34% No. (%)	*P*
Clinical characteristics						
Age (IQR), y	62 (58 to 66)	63 (59 to 67)	<.001	62 (58 to 66)	63 (59 to 67)	.005
Prior PSA screen	629 (21)	232 (13)	<.001	207 (17)	83 (13)	.024
Unknown	63 (2.1)	35 (2.0)		27 (2.2)	13 (2.0)	
Total PSA (IQR), ng/mL	4.3 (3.6 to 5.7)	5.4 (3.9 to 8.6)	<.001	4.5 (3.6 to 5.8)	5.6 (4.2 to 9.7)	<.001
Free PSA (IQR), ng/mL	1.00 (0.76 to 1.37)	0.97 (0.70 to 1.46)	.3	0.97 (0.71 to 1.35)	0.92 (0.68 to 1.46)	.7
Intact PSA (IQR), ng/mL	0.40 (0.27 to 0.58)	0.41 (0.26 to 0.66)	.095	0.38 (0.25 to 0.57)	0.43 (0.27 to 0.70)	<.001
hK2, ng/mL	0.043 (0.030 to 0.062)	0.049 (0.035 to 0.073)	<.001	0.041 (0.029 to 0.061)	0.053 (0.036 to 0.081)	<.001
Tumor characteristics						
Gleason sum score						
≤ 6		1099 (63)			464 (73)	
7		542 (31)			143 (22)	
≥ 8		92 (5.3)			32 (5.0)	
Stage						
T1		1016 (59)			330 (52)	
T2		331 (19)			88 (14)	
T3		127 (7.3)			63 (10)	
T4		4 (0.2)			2 (0.3)	
Unknown		255 (15)			156 (24)	

* The cohort underwent a 10-core prostate biopsy and had EDTA-anticoagulated plasma and/or serum available for retrospective measurements of total, free, and intact prostate-specific antigens (PSAs) and hK2, in cryopreserved sample aliquots. Data are median (interquartile range) or frequency (percentage). IQR = interquartile range; hK2 = human kallikrein-related peptidase 2; PSA = prostate-specific antigen.

The discriminatory accuracy of each combination of kallikrein markers, as measured in EDTA anticoagulated plasma, is outlined in Table 2. The discriminatory accuracy for the base model (age plus tPSA), as measured by the AUC, was 0.634 (95% confidence interval [CI] = 0.617 to 0.651) for any-grade prostate cancer. Adding fPSA, iPSA, and hK2 to this model improved the predictive accuracy (AUC 0.719 [95% CI = 0.704 to 0.734], increment 0.085, *P* < .001). Use of the base model to predict evidence of Gleason score 7 or higher (high-grade) prostate cancer at 10-core biopsy gave an AUC of 0.738 (95% CI = 0.716 to 0.761), while the additional kallikrein markers statistically significantly enhanced the AUC to 0.820 (95% CI = 0.802 to 0.838; increment 0.082, *P* < .001) (Table 2).

**Table 2.** Discriminatory accuracy of each kallikrein model*

Model	Plasma AUC (95% CI)	Increment over “Age + total PSA” (*P* value)
Any-grade prostate cancer		
Age + total PSA	0.634 (0.617 to 0.651)	
Age + total PSA and free-to-total PSA ratio	0.710 (0.695 to 0.725)	0.076 (*P* < .001)
Age + panel of four kallikrein markers	0.719 (0.704 to 0.734)	0.085 (*P* < .001)
High-grade prostate cancer		
Age + total PSA	0.738 (0.716 to 0.761)	
Age + total PSA and free-to-total PSA ratio	0.799 (0.779 to 0.819)	0.060 (*P* < .001)
Age + panel of four kallikrein markers	0.820 (0.802 to 0.838)	0.082 (*P* < .001)

* This table outlines the three combinations of markers for predicting any-grade or Gleason score 7 or higher (high-grade) cancer at 10-core prostate biopsy based on kallikrein marker measurements in anticoagulated plasma provided by 4765 biopsied ProtecT participants. Areas under the curve were compared with models based on standard clinically available predictors using the DeLong test (26). All statistical tests were two-sided. CI = confidence interval; PSA = prostate-specific antigen.

**Figure 1.**
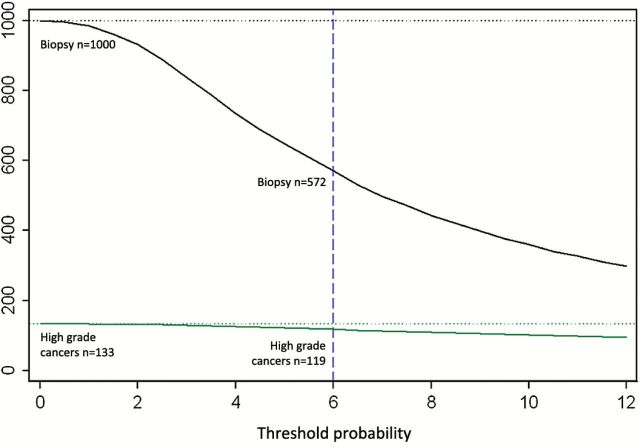
Clinical implications of various biopsy strategies using a model developed to predict the risk of Gleason score 7 or higher (high-grade) prostate cancer based on four kallikrein markers measured in anticoagulated plasma collected from 4765 biopsied ProtecT participants. The graph illustrates the results of differing biopsy strategies per 1000 biopsied ProtecT-participants, with the x-axis denoting the risk of high-grade cancer and the y-axis indicating the number of men biopsied (**black line**) or detected with evidence of high-grade cancer (**green line**) using different biopsy strategies. The **dotted vertical blue line** illustrates a tentative cutpoint (6% risk of high-grade cancer) at which only 572 of 1000 of the men would be biopsied, which would result in the detection of 119 of 133 high-grade cancers.

To explore the implications of using the models in clinical practice, we conducted a decision analysis to simulate outcomes if biopsy decisions had been based on various cutpoints from the model. The results are shown in Table 3 and Figure 1. For an illustrative threshold representing a 6% risk of Gleason score 7 or higher (high-grade) disease, use of this model would reduce the number of biopsies by 428 per 1000 biopsied men (43%), detect 119 high-grade cancers, and delay the diagnosis of 14 of 133 high-grade cancers, four of which would have had primary Gleason grade 4. A decision-curve analysis demonstrates that use of the model contributes added clinical value (Supplementary Figure 3, available online).

**Table 3.** Results of differing biopsy strategies per 1000 men screened at varying thresholds for risk of any-grade cancer or high-grade cancer among men with anticoagulated plasma

Threshold	Biopsies		Any-grade prostate cancer		Gleason score 7 or higher (high-grade)		Primary Gleason score 4 or higher*	
	Performed	Avoided (%)	Found	Delayed	Found	Delayed	Found	Delayed
Risk of any-grade cancer								
Biopsy all men	1000	0 (0)	364	0	133	0	47	0
Risk by age and total PSA								
≥20%	997	3 (0.3)	363	0	133	0	47	0
≥30%	642	358 (36)	264	99	111	22	41	6
Risk by age and panel of four kallikrein markers								
≥20%	834	166 (17)	334	30	129	4	46	1
≥30%	545	455 (46)	264	100	116	17	43	4
Risk of high-grade cancer								
Biopsy all men	1000	0 (0)	364	0	133	0	47	0
Risk by age and total PSA								
≥4%	974	26 (2.6)	357	7	132	1	47	0
≥6%	876	124 (12)	332	32	127	6	44	3
≥8%	715	285 (28)	284	79	117	16	42	5
≥10%	533	467 (47)	235	129	105	28	39	8
Risk by age and panel of four kallikrein markers								
≥4%	734	266 (27)	313	51	127	6	46	1
≥6%	572	428 (43)	270	94	119	14	43	4
≥8%	442	558 (56)	229	135	110	23	42	5
≥10%	361	639 (64)	203	161	103	30	39	8

* Includes cases with any Gleason Grade 5 component.

In a secondary analysis, we examined the properties of the four kallikrein markers measured in serum from another 1860 ProtecT participants (complete data shown in the Supplementary Materials, available online). Applying the previously reported “ERSPC-Rotterdam” (11) model improved the AUC compared with the base model (PSA plus age) from 0.665 to 0.709 (increment of 0.043, *P* = .010) for evidence of any-grade cancer at biopsy, and from 0.785 to 0.836 (increment of 0.052, *P* = .010) for high-grade cancer at biopsy. However, as the “ERSPC-Rotterdam” model underestimated risk for both endpoints (Supplementary Figure 1B, available online), and ERSPC used sextant biopsies in contrast to the minimum of a 10-core prostate biopsy used in ProtecT, we reestimated the coefficients of the kallikrein markers among men providing a serum sample in the ProtecT study and performed an internal validation of the updated model again utilizing 10-fold cross-validation. The AUC now improved from 0.665 to 0.757 (increment 0.092, *P* < .001) for evidence of any-grade cancer, and from 0.785 to 0.859 (increment 0.075, *P* < .001) for high-grade cancer as shown in Supplementary Table 2 (available online). Next, we evaluated whether the discriminatory properties of the kallikrein marker measurements were different in cryopreserved serum vs anticoagulated plasma using paired serum and plasma samples from 496 ProtecT participants (Supplementary Table 3, available online). We found no differences in the predictive accuracy of the markers measured in serum compared with plasma.

Finally, as an external validation of the model developed to predict high-grade cancer based on kallikrein markers measured in anticoagulated plasma from 4765 biopsied ProtecT participants, we assessed the discriminatory accuracy of this model using 1364 biopsied ProtecT participants with only serum samples available to measure the four kallikrein markers and excluding 496 ProtecT participants with both anticoagulated plasma and serum samples available. Using this independent group of 1364 biopsied ProtecT participants, our external validation showed that the model exhibits excellent discriminatory accuracy (AUC = 0.849, 95% CI = 0.814 to 0.883) (Supplementary Data Section 5, available online).

## Discussion

In this study we demonstrate that a panel of four kallikrein markers—total PSA, free PSA, intact PSA, and hK2—can predict the result of prostate biopsy. A statistical model based on the four markers improved this prediction above and beyond both PSA and age, as well as beyond a combination of total and free PSA (Table 2). A decision analysis indicated that use of the statistical model to guide biopsy decisions would reduce the number of men receiving unnecessary biopsies, without substantially affecting the diagnosis of Gleason score 7 or higher (high-grade) cancers.

Two initial studies on the Göteborg arm of the ERSPC demonstrated that the panel of markers improved prediction of biopsy outcome for both unscreened men (10) and those with a previous PSA in the normal range (28). Our results confirm these findings. The assays were subsequently modified, and a new statistical model was built based on a group of unscreened men in the Rotterdam arm of ERSPC. This was termed the “Rotterdam” model and was shown to improve the prediction of biopsy outcome over and above PSA in several independent validation cohorts, including unscreened men (11,14), men with prior screening (29), those with prior negative biopsy (13), and those subject to clinical work-up before biopsy (12). The AUC results in these previous studies are very similar to those found here, including an AUC of 0.820 for high-grade cancer, representing an increment of 0.082 over age and PSA alone. For instance, in unscreened men in ERSPC, we reported an AUC of 0.825, 0.049 higher than the base model (11), and for previously screened men the AUC was 0.793, an increment of 0.094 beyond the base model (29).

The Rotterdam model was also shown to predict clinical cancer endpoints in men who were never screened from the Malmö Diet and Cancer study, a population-based cohort of 11 063 Swedish men aged 45 to 73 years who provided blood between 1991 and 1996. Subsequent diagnosis of prostate cancer was assessed by linking with the Swedish Cancer Registry, updated to the end of 2006, and this occurred in 943 men. Rates of PSA screening were very low in Sweden during the study. The Rotterdam model was applied to a subset of men with a total PSA level 3.0ng/mL or more at baseline. The concordance index for clinical diagnosis of cancer was higher for the Rotterdam model compared with PSA alone (0.65 vs 0.75, *P* < .001). For every 1000 men with a total PSA level 3ng/mL or more at baseline, the model would classify as high-risk 131 of 152 (86%) of the cancer case patients diagnosed clinically within five years, and 421 men would be classified as low-risk by the panel and recommended to forgo biopsy. Of these, only two men would be expected to be diagnosed with advanced prostate cancer (clinical T3 to T4 or metastases) within five years. Hence, while some men with a low score on the Rotterdam model including the four kallikrein markers do indeed have biopsy-detectable cancer, only a very small proportion have aggressive disease that would become apparent over time.

The current study goes above and beyond the previous research not only in terms of size—with a sample size approaching all previous papers on the kallikrein markers combined—but also in the use of plasma samples, which appear to be more appropriate for analysis in clinical care, as the biomarkers are less prone to decay in plasma compared with serum samples. Moreover, patients in the study underwent a minimum of 10 core biopsies, rather than the outdated sextant biopsy used in previous studies. Our study also provides clear evidence that the statistical model based on the four kallikrein markers could be used in everyday clinical practice in order to aid decisions about prostate biopsy.

The study has a number of limitations that would need to be addressed in subsequent research. First, fresh samples would need to be assayed in a routine clinical laboratory rather than in a research setting using cryopreserved samples. Second, the ProtecT trial protocol offered biopsies to all men with PSA levels of 3ng/mL or greater, and notably 87% of men accepted the offer of a biopsy. In routine clinical practice, a man presenting to a urologist with an elevated PSA would be subjected to clinical work-up, including assessment of benign disease and frequently a repeat PSA (30). Biopsy might not be indicated if the PSA is lower on repeat testing or if the PSA elevation is attributed to a benign cause. It may be that some of the information provided by the kallikrein model is already captured in such a clinical work-up. If so, it is plausible that while the kallikrein model may be of value where all men with elevated PSA are biopsied, it is not of value for men selected for biopsy according to a urologist’s clinical judgment. Third, the ProtecT cohort does not reflect clinical practice in the United Kingdom, where little opportunistic PSA testing takes place in the community (31) in contrast with many other countries where PSA testing and retesting prevails.

A definitive four-kallikrein panel evaluation will require the prospective analysis of samples taken from men with elevated PSA levels in whom urologists have made the clinical judgment to perform a biopsy. The cohort should include all-comers, irrespective of prior screening, PSA cutoff used, or biopsy history. Kallikrein markers should be measured from plasma in a clinical laboratory within one or two days of the blood draw. The biopsy would involve the 12 or 14 core approaches common in US practice. Marker values should be incorporated into a prespecified statistical model including data on digital rectal examination and history of prior biopsy. Such studies are currently underway.

In conclusion, we provide evidence that a panel of four kallikrein markers, incorporating total PSA, free PSA, intact PSA, and hK2, is superior to total PSA alone in predicting the result of prostate biopsy. The markers differentially detected high-grade disease. In a decision analysis we found that implementation of a statistical model based on the markers would reduce by close to half the number of unnecessary biopsies undertaken, while delaying diagnosis of only a small number of high-grade cancers. These findings need to be confirmed in prospective research using clinical cohorts.

## Funding

This work is supported by the National Cancer Institute at the National Institutes of Health (R01 CA160816 to HL and AV, P50 CA092629 to Craig Thompson, and R01 CA175491 to Robert Klein), the Sidney Kimmel Center for Prostate and Urologic Cancers, David H. Koch through the Prostate Cancer Foundation, the National Institute for Health Research (NIHR) Oxford Biomedical Research Centre Program, the Cancer Research UK Oxford Centre, the Swedish Cancer Society (project no. 11–0624 to HL), a FiDIPro-program award to HL from TEKES, and Fundacion Federico SA. The ProtecT study is funded by the UK National Institute for Health Research Health Technology Programme (96/20/99).

## Supplementary Material

Supplementary Data

## Notes

Author Contributions: Dr. Lilja had full access to all of the data in the study and takes responsibility for the integrity of the data and the accuracy of the data analysis. Study concept and design: HL and AJV. Acquisition, analysis, or interpretation of data: HL, RB, LM, RK, MD, FCH, DDS, MCR, and AJV. Drafting of the manuscript: RB, DDS, AJV, and HL. Statistical analysis: DDS and AJV. Study supervision: AJV, FCH, and HL. FCH, JD, and DEN are Principal Investigators of the NIHR HTA ProtecT study.

Hans Lilja holds patents for free PSA, hK2, and intact PSA assays, and is named, along with Andrew J. Vickers, on a patent application for a statistical method to detect prostate cancer. The marker assay patents and the patent application for the statistical model has been licensed and commercialized as the 4K score by OPKO Diagnostics. Dr. Vickers, Scardino, and Lilja may receive royalties from sales of this test. Additionally, Drs. Scardino and Lilja own stock and Dr. Vickers owns stock options in OPKO.

We are grateful to Gemma Marsden and Athene Lane for their help with this project and to the ProtecT pathology group: Jon Oxley (Chair), John Goepel, Murali Varma, David Griffiths, Ken Grigor, Nick Mayer, Ann Warren, Nayneeta Deshmukh, Selina Battharai, John Dormer, and L. A. Adamczyk. We thank Gun-Britt Eriksson, Mona Hassan Al-Battat, and AnnaPeri Erlandsson for expert assistance with immunoassay measurements of the blood samples at the Wallenberg Research Laboratories at Lund University in Malmö, Sweden.
