# Supplementary material for: Predicting High-Grade Cancer at Ten-Core Prostate Biopsy Using Four Kallikrein Markers Measured in Blood in the ProtecT Study
Source: J Natl Cancer Inst. 2015 Apr 11;107(7):djv095. doi: 10.1093/jnci/djv095 (PMC4554254; doi:10.1093/jnci/djv095)
Supplement: Supplementary Data [file supp_107_7_djv095__index.html]

Predicting High-Grade Cancer at Ten-Core Prostate Biopsy Using Four Kallikrein Markers Measured in Blood in the ProtecT Study — Supplementary Data 

# Predicting High-Grade Cancer at Ten-Core Prostate Biopsy Using Four Kallikrein Markers Measured in Blood in the ProtecT Study

## Supplementary Data

Data files

**Files in this Data Supplement:**

- Supplementary Data - Supplementary Data
