# Supplementary material for: Predicting High-Grade Cancer at Ten-Core Prostate Biopsy Using Four Kallikrein Markers Measured in Blood in the ProtecT Study
Source: J Natl Cancer Inst. 2015 Apr 11;107(7):djv095. doi: 10.1093/jnci/djv095 (PMC4554254; doi:10.1093/jnci/djv095)
Supplement: Supplementary Data [file supp_djv095_Supplementary_Materials.pdf]

Supplementary Materials

Supplementary Data

1. Analysis of the potential effects of missing samples on the overall performance of the four kallikrein models.

At the inception of the ProtecT study blood samples were not uniformly collected and stored, and therefore the kallikrein markers are not available for all men who underwent a biopsy. Even after blood samples began to be stored, not all centres uniformly stored samples for all men who underwent biopsy. While 7,471 men underwent biopsy, 6,129 biopsied men (82%) had accessible samples, with EDTA anti-coagulated plasma retrieved from 4,765 of these men. The observed pattern of missing samples can be explained by the gradual implementation of standardized research sample collection protocols after the commencement of recruitment to ProtecT, with only serum samples saved during the first few years of the study. However, as the protocols for saving samples became standardized across participating centers, the number of men with stored plasma samples increased and as both serum and plasma were stored from some participants, we had a unique opportunity to compare directly whether there was a difference in predictive discrimination of the panel in each sample type.

We noted that among men for whom a plasma sample was available, the rate of positive biopsy was similar compared with all men who underwent a prostate biopsy (36% and 35% respectively for any-grade cancer, and 12% and 13% for men with evidence of Gleason score 7 or higher (high-grade) disease).

As there are a large proportion of men for whom we do not have a blood sample, we wished to assess whether this missing data had an effect on our results. We therefore built a logistic regression model to predict cancer on biopsy using age and the kallikrein markers with data from three centers with the fewest missing blood samples, and we applied this model to the data collected from the remaining six centers. The discrimination of the model was nearly identical for the centers with the fewest missing blood samples and the other six centers (difference in AUC is less than 0.01), suggesting the missing samples did not affect our results.

2. Analysis of the use of the Rotterdam serum-based model in ProtecT plasma and serum samples.

We initially analyzed the performance of the previous "Rotterdam model", developed using the Rotterdam screening arm cohort of the European randomized screening trial (ERSPC) study (1), in the 496 ProtecT study men with paired plasma and serum samples. The characteristics of these 496 men are shown in Supplementary table 1. We wondered whether the performance of the Rotterdam model in ProtecT samples might be influenced by factors that were different in ProtecT compared with ERSPC. For example, the Rotterdam model had been built using serum-based measurements, and the ERSPC had used a sextant prostate biopsy protocol. We were interested to see how the previous ERSPC-Rotterdam model might perform when it was applied to our contemporary ProtecT study cohort, which used an extended ten-core prostate biopsy protocol, a factor which at least in part explains the 35% rate of any-grade prostate cancer detection in ProtecT versus 25% in Rotterdam ERSPC (2). We were also interested to see how the Rotterdam model would perform for plasma-based samples, given that at least some of the kallikrein markers are more labile in serum than plasma. Finally, we wondered whether the rate of prior PSA testing in our ProtecT study men might influence the performance of the model.

We tested the ability of the Rotterdam serum-based model to predict the presence of any-grade prostate cancer at biopsy using ProtecT plasma and serum samples. When the Rotterdam model was applied to the 496 men with paired samples we observed an enhancement in predicting biopsy outcome compared with the use of PSA alone. However, we also saw a considerable degree of mis-calibration of the Rotterdam model when it was applied to ProtecT samples, particularly in the case of plasma samples (Supplementary Figure 1).

3. Analysis of the use of the model in serum samples.

We investigated the performance of our new serum-based ProtecT model in predicting any-grade and high-grade prostate cancer in biopsied men with a serum sample available for analysis. The demographics of men with a serum sample are shown in table 1. We observed an important difference between the cohorts of men with either plasma or serum samples in terms of high-grade cancer, with 13% of 4,765 biopsied men with a plasma sample having high-grade disease versus 9% for 1,860 biopsied men with a serum sample. The base model (age plus tPSA) had a relatively poor predictive accuracy for diagnosing any-grade prostate cancer at biopsy for men with serum samples, with an AUC of 0.665 (Supplementary table 2). Adding fPSA, iPSA, and hK2 to the new serum model significantly improved its predictive accuracy, resulting in an AUC of 0.757 (increment of 0.092, $P < .001$). Application of the new base model to the detection of high-grade cancers resulted in an AUC of 0.785 for serum samples (Supplementary table 2), and the inclusion of additional kallikreins as a "full" model significantly enhanced the AUC for high-grade prostate cancer (0.859 for serum, an increment of 0.075, $P < .001$). Importantly, when we tested the 496 paired plasma and serum samples we observed no significant difference in the performance of the model in predicting any-grade or high-grade prostate cancer at biopsy using four kallikrein markers measured in plasma versus serum (Supplementary table 3 and Supplementary figure 2). Applying a ≥20% risk scenario for the detection of any-grade prostate cancer to the serum-based kallikrein-marker model would eliminate 301 biopsies per 1,000 currently biopsied men, detect 299 cancers and delay the diagnosis of 44 prostate cancers (3 Gleason Score 7 or higher cancers, no Gleason primary grade 4) (see Supplementary table 4). A ≥30% risk scenario applied to serum would eliminate 529 biopsies per 1,000 biopsied men, and

detect 249 cancers while delaying the diagnosis of 95 cancers (8 with Gleason Score 7 or higher cancers, of which one was a primary Gleason grade 4 cancer).

4. Marginal value of kallikrein markers.

We sought to investigate the added value of each of the kallikrein markers for predicting high grade and any grade cancer on biopsy. To assess the marginal value of the kallikrein markers we re-fit the logistic regression models omitting each of the markers (the full model includes age, total PSA, free PSA, intact PSA, and hK2). Ten-fold cross validation was utilized to correct the area under the curve (AUC) estimates for overfit. As expected, when total PSA was omitted from the model the AUC decreased by the greatest magnitude. For the outcome of high grade cancer, the AUC fell from 0.820 to 0.703 among patients with a blood plasma measurement and from 0.859 to 0.765 among patients with a blood serum measurement for high grade cancer (Supplementary Table 5). When intact PSA and hK2 were omitted from the model the AUC decreased by 0.02 among both blood plasma and blood serum cohorts for high grade cancer, suggesting an improvement in model performance when including intact PSA and hK2 over total and free PSA alone.

5. Further Validation of Kallikrein Predictive Models.

The kallikrein models discriminate well between men with and without high grade cancers, and consequently many men with low grade disease or without cancer can avoid prostate biopsy. These results are based on an internal 10-fold cross validation of the predictive models. However, external validation is the most robust method for assessing a model’s performance, where none of the data used to build a model are utilized in evaluating its performance. To this end, we assessed the discrimination of the kallikrein model built on the 4,765 men who provided a plasma blood sample on 1,364 who provided a serum sample excluding 496 men with both plasma and serum blood samples available. Similarly, we assessed the performance of the model

built on the 1,860 men who provided a serum sample by applying it to the 4,269 men with a plasma sample and no serum sample. While we do expect some miscalibration due the differing properties of serum and plasma kallikrein measurements, discrimination is robust to miscalibration. Again, we observed on external validation that both models exhibit excellent discrimination. The AUC when applying the plasma-based kallikrein model to the patients with serum samples was 0.849 (95% CI = 0.814 to 0.883), which is slightly lower than observed after internal 10-fold validation, where the serum-based model had an AUC of 0.859 (95% CI = 0.830 to 0.888). The AUC of 0.773 (95% CI = 0.752 to 0.794) for applying the serum-based kallikrein model to men with a plasma sample was also slightly lower than what was observed after internal 10-fold validation, where the plasma-based model had an AUC of 0.820 (95% CI = 0.802 to 0.838).

Supplementary Figure 1. Calibration of the Rotterdam model applied to the ProtecT biopsy cohort.

A) Plasma

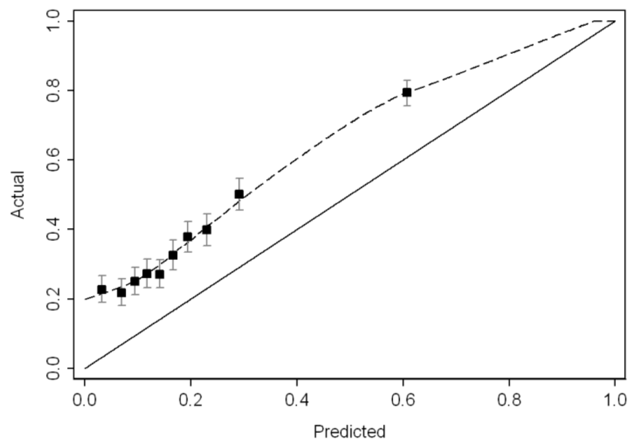

B) Serum

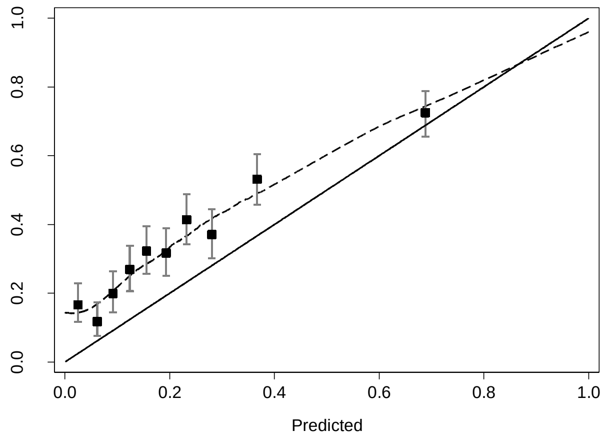

Supplementary Figure 2. Clinical implications of various biopsy strategies. This graph is based on a model developed to predict risk of Gleason score 7 or higher (high-grade) prostate cancer using kallikrein markers measured in serum collected from 1,860 biopsied ProtecT participants.

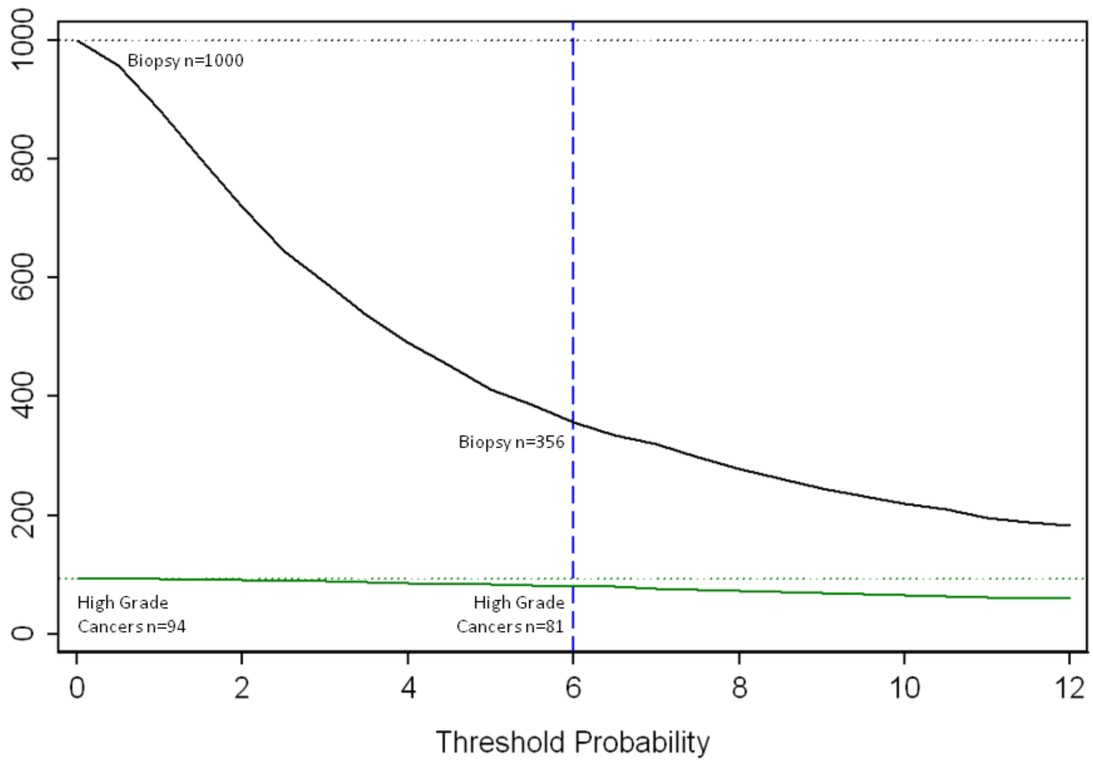

Supplementary Figure 3. Decision curve analysis. Age and four kallikrein markers are represented by the solid black line, age and PSA alone are represented by the grey line, benefit of biopsying all men is indicated by the dashed black line, and the dashed grey line indicates net benefit of biopsying no men.

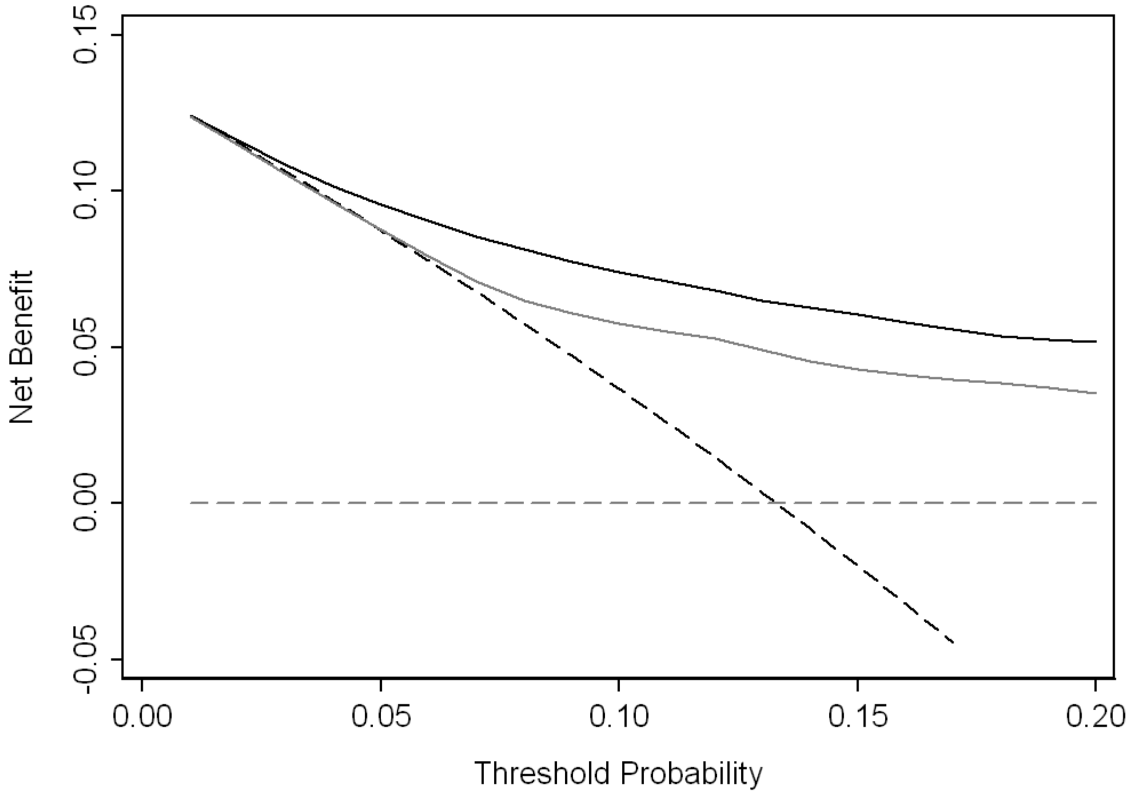

Supplementary Table 1. Characteristics of the 496 men in the ProtecT study cohort who underwent a prostate biopsy and had both plasma and serum samples available for analysis.

	Data for men with paired plasma and serum samples		
	**No cancer detected (n=351; 71%)**	**Diagnosed with cancer (n=145; 29%)**	***P* value**
**Clinical characteristics**			
Age (years)	62 (58 to 66)	64 (60, 67)	.005
Prior PSA Screen	72 (21%)	20 (14%)	.077
Unknown	8 (2.3%)	3 (2.1%)	
**Plasma samples**			
Total PSA (ng/mL)	4.3 (3.5 to 5.5)	5.9 (4.0 to 10.4)	< .001
Free PSA (ng/mL)	0.97 (0.74 to 1.31)	1.09 (0.78 to 1.59)	.022
Intact PSA (ng/mL)	0.36 (0.25 to 0.52)	0.44 (0.25 to 0.72)	.006
hK2 (ng/mL)	0.040 (0.029 to 0.055)	0.050 (0.037 to 0.077)	< .001
**Serum samples**			
Total PSA (ng/mL)	4.1 (3.4 to 5.4)	5.4 (3.9 to 9.7)	< .001
Free PSA (ng/mL)	0.96 (0.71 to 1.26)	1.10 (0.75 to 1.56)	.024
Intact PSA (ng/mL)	0.44 (0.34 to 0.67)	0.57 (0.40 to 0.80)	.001
hK2 (ng/mL)	0.039 (0.027 to 0.055)	0.054 (0.037 to 0.082)	< .001
**Tumour characteristics**			
Gleason Sum Score			
≤ 6		94 (65%)	
7		42 (29%)	
≥ 8		9 (6.2%)	
Stage			
T1		76 (52%)	
T2		30 (21%)	
T3		11 (7.6%)	
T4		0 (0%)	
Unknown		28 (19%)	

Data are median (interquartile range) or frequency (percentage). PSA = prostate-specific antigen; hK2 = human kallikrein peptidase 2.

Supplementary Table 2. Discriminatory accuracy of each kallikrein marker*

**Model**	**Any-grade prostate cancer**	
	**Serum AUC (95% CI)**	**Increment over "Age + total PSA" (*P* value)**
Age + total PSA	0.665 (0.639 to 0.692)	
Age + total PSA and free-to-total PSA ratio	0.741 (0.717 to 0.765)	0.075 (*P* < .001)
Age + panel of four kallikrein markers	0.757 (0.734 to 0.780)	0.092 (*P* < .001)
ERSPC-Rotterdam four kallikrein model	0.709 (0.684 to 0.734)	0.043 (*P* = .010)
**Model**	**High-grade prostate cancer**	
	**Serum AUC (95% CI)**	**Increment over "Age + total PSA"**
Age + total PSA	0.785 (0.745 to 0.824)	
Age + total PSA and free-to-total PSA ratio	0.839 (0.808 to 0.871)	0.055 (*P* < .001)
Age + panel of four kallikrein markers	0.859 (0.830 to 0.888)	0.075 (*P* < .001)
ERSPC-Rotterdam four kallikrein model	0.836 (0.803 to 0.869)	0.052 (*P* = .010)

* This table outlines the various combinations of markers for predicting any-grade and Gleason score 7 or higher (high-grade) prostate cancer for 1,860 biopsied men with four kallikrein markers measured in serum. PSA = prostate-specific antigen; AUC = area under the curve; CI = confidence interval

Supplementary Table 3. Results from differing biopsy strategies per 1,000 men screened at varying thresholds for risk of any-grade cancer (panel A) and high-grade cancer (panel B) among men with serum.

A) Risk of any grade cancer

	**Biopsies**		**Any-grade prostate cancer**		**Gleason Score 7 or higher (High-grade)**		**Primary Gleason Score 4 or higher***	
	**Performed**	**Avoided (%)**	**Found**	**Delayed**	**Found**	**Delayed**	**Found**	**Delayed**
Biopsy All Men	1,000	0 (0%)	344	0	94	0	34	0
Risk by age and total PSA								
≥20%	897	103 (10%)	327	16	94	0	34	0
≥30%	575	425 (43%)	242	101	79	15	31	3
Risk by age and panel of four kallikrein markers								
≥20%	699	301 (30%)	299	44	91	3	34	0
≥30%	471	529 (53%)	249	95	87	8	33	1

* Includes cases with any Gleason Grade 5 component.

B) Risk of high grade cancer

	**Biopsies**		**Any-grade prostate cancer**		**Gleason Score 7 or higher (High-grade)**		**Primary Gleason Score 4 or higher***	
	**Performed**	**Avoided (%)**	**Found**	**Delayed**	**Found**	**Delayed**	**Found**	**Delayed**
Biopsy All Men	1,000	0 (0%)	344	0	94	0	34	0
Risk by age and total PSA								
≥4%	724	276 (28%)	289	55	87	7	32	2
≥6%	560	440 (44%)	236	108	78	16	30	4
≥8%	349	651 (65%)	169	175	68	26	25	9
≥10%	204	796 (80%)	117	227	55	39	23	11
Risk by age and panel of four kallikrein markers								
≥4%	490	510 (51%)	249	95	87	8	33	1
≥6%	356	644 (64%)	208	135	81	13	31	3
≥8%	278	722 (72%)	173	171	73	21	29	5
≥10%	219	781 (78%)	148	196	66	28	26	8

* Includes cases with any Gleason Grade 5 component.

Supplementary Table 4. A comparison of the accuracy to predict any-grade or Gleason score 7 or higher (high-grade) prostate cancer on biopsy in 496 biopsied ProtecT participants who had both EDTA anti-coagulated plasma and serum samples available for analysis.

	**Any-grade prostate cancer**		
**Model**	**Plasma AUC (95% CI)**	**Serum AUC (95% CI)**	***P* value comparing AUCs**
Age + total PSA	0.691 (0.637 to 0.745)	0.684 (0.630 to 0.737)	.4
Age + total PSA and free-to-total PSA ratio	0.726 (0.673 to 0.778)	0.725 (0.673 to 0.777)	1
Age + panel of four kallikrein markers	0.748 (0.697 to 0.799)	0.746 (0.694 to 0.797)	.9
	**High-grade prostate cancer**		
**Model**	**Plasma AUC (95% CI)**	**Serum AUC (95% CI)**	***P* value comparing AUCs**
Age + total PSA	0.831 (0.764 to 0.899)	0.830 (0.761 to 0.900)	.9
Age + total PSA and free-to-total PSA ratio	0.853 (0.794 to 0.912)	0.853 (0.791 to 0.914)	1
Age + panel of four kallikrein markers	0.890 (0.842 to 0.939)	0.884 (0.827 to 0.941)	.6

PSA = prostate-specific antigen; AUC = area under the curve; CI = confidence interval

Supplementary Table 5. Marginal improvement in discrimination (AUC) for each kallikrein marker.

A) Plasma

	Any Grade Cancer	High Grade Cancer
Full Model	0.719 (0.704, 0.734)	0.820 (0.802, 0.838)
Without tPSA	0.635 (0.619, 0.652)	0.703 (0.703, 0.725)
Without fPSA	0.684 (0.668, 0.700)	0.753 (0.731, 0.776)
Without iPSA and hK2	0.709 (0.693, 0.724)	0.801 (0.781, 0.821)

B) Serum

	Any Grade Cancer	High Grade Cancer
Full Model	0.757 (0.734, 0.780)	0.859 (0.830, 0.888)
Without tPSA	0.669 (0.643, 0.694)	0.765 (0.726, 0.803)
Without fPSA	0.686 (0.660, 0.712)	0.788 (0.749, 0.828)
Without iPSA and hK2	0.743 (0.719, 0.767)	0.839 (0.807, 0.871)

References

1. Benchikh A, Savage C, Cronin A, et al. A panel of kallikrein markers can predict outcome of prostate biopsy following clinical work-up: an independent validation study from the European Randomized Study of Prostate Cancer screening, France. *BMC Cancer*. 2010;10:635.
2. Schroder FH, Hugosson J, Roobol MJ, et al. Screening and prostate-cancer mortality in a randomized European study. *N Engl J Med*. 2009;360(13):1320-1328.
